# Changes in the Management of Patients with Crohn's Disease Based on Magnetic Resonance Enterography Patterns

**DOI:** 10.1155/2019/3467316

**Published:** 2019-12-17

**Authors:** Evelyn Sayuri S. Chinem, Barbara C. Esberard, Andre da L. Moreira, Tatiana G. Barbassa, Guilherme M. da Cunha, Antonio Jose de V. Carneiro, Heitor S. de Souza, Ana Teresa P. Carvalho

**Affiliations:** ^1^Disciplina de Gastroenterologia e Endoscopia Digestiva, Universidade do Estado do Rio de Janeiro, Rio de Janeiro, RJ 20551-900, Brazil; ^2^Serviço de Gastroenterologia do Hospital Universitário Gaffree e Guinle, Rio de Janeiro, RJ 20270-901, Brazil; ^3^Disciplina de Proctologia, Universidade do Estado do Rio de Janeiro, Rio de Janeiro, RJ 20551-900, Brazil; ^4^CDPI/DASA (Clínica de Diagnoóstico por Imagem/Diagnóstico das Américas S/A), Rio de Janeiro, RJ 22440-032, Brazil; ^5^Serviço de Gastroenterologia, Departamento de Clínica Médica, Universidade Federal do Rio de Janeiro, Rio de Janeiro, RJ 21941-913, Brazil

## Abstract

**Background and Aims:**

Magnetic resonance enterography (MRE) has become an important modality of radiological imaging in the evaluation of Crohn's disease (CD). The aim of this study was to investigate the impact of MRE in the assessment of disease activity and abdominal complications and in the making of therapeutic decisions for patients with CD.

**Methods:**

In a cross-sectional retrospective study, we selected 74 patients with CD who underwent MRE and ileocolonoscopy with an interval between the two exams of up to 30 days between January 2011 and December 2017. We assessed the parameters of the images obtained by MRE and investigated the agreement with the level of disease activity and complications determined by a clinical evaluation, inflammatory biomarkers, and endoscopy, as well as the resulting changes in medical and surgical management.

**Results:**

Changes in medical management were detected in 41.9% of patients. Significant changes in medical decisions were observed in individuals with a purely penetrating (*P* = .012) or a mixed (*P* = .024) MRE pattern. Patients with normal MRE patterns had a correlation with unchanged medical decisions (*P* = .001). There were statistically significant agreements between the absence of inflammatory criteria on MRE and remission according to the Harvey and Bradshaw index (HBI) (*P* = .037), the presence of inflammatory criteria on MRE and positive results for calprotectin (*P* = .005), and penetrating criteria on MRE and the scoring endoscopic system for Crohn's disease (SES-CD), indicating active disease (*P* = .048). Finally, there was significant agreement between the presence of fibrostenotic criteria and a long disease duration (*P* = .027).

**Conclusion:**

MRE discloses disease activity and complications not apparent with other modalities and results in changes in therapeutic decisions. In addition to being used for diagnosis, MRE should be routinely used in the follow-up of CD patients.

## 1. Introduction

Crohn's disease (CD) is a chronic inflammatory condition of the gastrointestinal tract representing one of the two forms of inflammatory bowel disease (IBD). CD has been considered a clinically heterogeneous disease with variable degrees of severity and an unpredictable course [1]. Although marked advances have been achieved recently in the field of disease pathogenic mechanisms [2, 3], CD remains incurable, and the outcomes of patients are frequently unsatisfactory. The objective evaluation of disease activity, the detection of complications and changes in disease behaviour, and the response to treatment continue to be rather challenging. Nevertheless, given the current therapeutic arsenal available, the precise assessment of CD features has become increasingly important for the tailored and optimized care of patients, with the expectation of modifying the natural course of CD [4, 5].

In terms of diagnosis and follow-up, several methods have been proposed to assess CD characteristics, including disease activity and complications, but a consensus regarding the best exam has yet to be reached [6]. Endoscopy has been regarded as a mainstay method in both the diagnosis and follow-up of patients, but it is invasive, not free from risk, and may be subject to limitations depending on the disease location [7, 8]. In fact, the small bowel is affected in the majority of cases of CD, and therefore, its evaluation is of utmost importance in the diagnosis and follow-up of patients [9]. Radiological exams are more often used in this context because traditional endoscopic exams do not reach this region in the gastrointestinal tract.

For many years, the only radiological imaging available for the evaluation of patients with CD was small bowel follow-through (SBFT) with barium contrast. More recently, computed tomography (CT), magnetic resonance imaging (MRI), and ultrasound have been frequently used. Studies based on imaging methods show that computed tomography enterography (CTE), magnetic resonance enterography (MRE), and bowel ultrasound have similar levels of accuracy for diagnosis [10] and the evaluation of inflammatory disease activity [11], with radiological imaging recommended for small bowel assessment in the European Crohn's and Colitis Organization's guidelines [12]. Regarding the specific features of the imaging exams, MRI shows a higher tissue contrast, allowing a better characterization of the inflammatory and fibrotic changes in the intestinal wall [13], and may be superior to CT in the detection of stenosis [14]. Another important and well-known advantage of MRI is the absence of ionizing radiation [14]. Although under optimal conditions bowel ultrasound and MRE may provide comparable results, dependence on the operator experience with ultrasound has favoured MRE, when available [15].

Cross-sectional imaging provides information about the topography, extent, mucosal disease activity, and intra- and extraluminal complications, such as stenosis, abscesses, fistulae, and perforation. Nevertheless, studies show that CTE involves much higher doses of radiation compared with SBFT [16]. The relapsing and remitting nature of CD frequently exposes patients to repeated imaging studies of the bowel, leading to significant cumulative radiation exposure. Several characteristics of patients with CD, such as a young age at diagnosis, upper gastrointestinal involvement, penetrating disease, a history of multiple surgeries, and treatment with corticosteroids, have been associated with an increased likelihood of repetitive imaging tests [17, 18]. Cumulative high radiation exposure was defined as a cumulative effective dose (CED) greater than 75 millisieverts, equivalent to 3750 chest radiographs. The cumulative exposure to ionizing radiation of this magnitude was previously estimated to be responsible for increasing cancer mortality by 7.3% [19]. For patients with CD, MRI offers an alternative that does not involve exposure to ionizing radiation. However, MRI has some disadvantages, such as low spatial resolution, a long scanning time, and high costs. Due to the lack of ionizing radiation and increased tissue contrast, MRI has become an important radiological examination modality in the evaluation of CD.

Although various MRI findings have been proposed as potential imaging biomarkers of CD activity, more studies are still necessary to determine the correlation of imaging parameters with disease manifestations and the clinical indices used to assess CD. However, few studies have evaluated the impact of MRI findings on patient therapeutic management and which radiological parameters correlated better with such changes [20–22]. Moreover, the studies found in the literature are mostly small, retrospective, and unicentric. The aims of this study were to correlate the radiological changes found in the MRE images of patients with CD with several clinical, laboratory, and endoscopic parameters and to evaluate the impact of MRE on the modification of medical treatment.

## 2. Patients and Methods

### 2.1. Ethical Considerations

The Ethics Committee of the University Hospital of the State University of Rio de Janeiro approved the study protocol (CAAE: 61935315.4.0000.5259), which was implemented in agreement with the ethical standards described in the 1964 Declaration of Helsinki.

### 2.2. Patient Cohort

This cross-sectional study was carried out at the University Hospital of the State University of Rio de Janeiro (a tertiary care setting), where the medical records of patients with CD routinely followed up in the outpatient unit were retrospectively evaluated. Archival data between January 2011 and December 2017 were considered for inclusion in the study whenever patients had undergone MRE and ileocolonoscopy within an interval of 30 days. Patients with a history of previous malignant neoplasia or acquired immunodeficiency syndrome were excluded from the study.

A review of the medical records included the following data registered during the consultation at which MRE was requested: sex, age at diagnosis, phenotype and location of the disease (Montreal classification), history of smoking, presence of extraintestinal manifestations, history of surgeries due to CD, drug therapy, Harvey and Bradshaw Index (HBI), haemoglobin level, C-reactive protein (CRP) level, erythrocyte sedimentation rate (ESR), ileocolonoscopy, and indications for MRE (symptomatic patients, evaluation of the extent of disease in the small bowel, assessment before biological therapy or surgery, and assessment of the response to drug therapy). The modification of medical therapy within an interval of 30 days of MRE was also recorded. Management changes owing to the MRE results were divided into the following groups: unchanged, optimization of drug treatment (increase in the current drug dose, the addition of another drug, or a change of drug in the therapeutic regimen), reduction in drug treatment (reduction in the drug dose or the suspension of medication), surgery, and indications for antibiotic therapy followed or not by abscess drainage. The clinical activity of the disease was classified according to the HBI into clinical remission (<5), mild disease (5–7) and moderate/severe disease (>7) [23]. Ileocolonoscopies were classified according to the simple endoscopic score for Crohn's disease (SES-CD) into endoscopic remission (0-2), mild endoscopic activity (3–6), moderate endoscopic activity (7–15), and severe endoscopic activity (>15). For patients who underwent ileocolonic surgery due to CD, ileocolonoscopies were classified according to the Rutgeerts score into remission (i0-i1) and endoscopic activity (i2-i4).

### 2.3. MRI

Prior to MRE acquisition, 20 mg of metoclopramide was injected intravenously, followed by the oral administration of a solution containing water and 2.5% mannitol. Approximately 1500 mL of the solution was administered for 30 min prior to the examination at a rate of approximately 200 mL every 5 min.

Images of the abdomen were acquired with the patient in supine position using the following sequences: axial and coronal half-Fourier acquisition single-shot turbo spin-echo (HASTE) with a 4 mm thickness, with and without fat suppression, and true fast imaging with steady state precession (TRUFI) with a 6 mm thickness. T1-weighted images (T1W 3D GRE) with fat suppression in the coronal plane (fat-saturated volume interpolated breath-hold images) were acquired before and after the administration of gadolinium through a dynamic study that included arterial (25-30 seconds after contrast injection), portal (80 seconds), and delayed (180 seconds) imaging. Immediately prior to the acquisition of T1-weighted images with coronal fat suppression, intravenous antispasmodic medication (scopolamine) was administered to reduce imaging artefacts due to intestinal peristalsis. Two board-certified radiologists (G.M. and T.B.) with more than twenty years of experience in IBD analysed all the MRE exams.

To search for possible associations between patient characteristics and changes in medical management based on the MRE results, patients were classified into one of the four disease patterns: inflammatory, fibrostenosing, penetrating or mixed type, or no abnormality. The criteria for the inflammatory pattern included early mucosal enhancement, mesenteric fat infiltration, lymphadenopathy, and engorgement of the mesenteric veins (comb sign). The criteria for the fibrostenosing pattern included fixed stenosis, parietal thickening, and upstream dilatation. The criteria for the penetrating pattern included abscesses and fistulas. Patients with criteria for two or more patterns were classified as having a mixed type.

To analyse the agreement of the MRE parameters with selected clinical, endoscopic, and laboratory variables, patients were divided into three major categories: inflammatory, penetrating, or fibrostenosing.

### 2.4. Statistical Analysis

Statistical analysis was performed using SPSS statistical software for Windows (Version 20, SPSS, Inc., Chicago, IL, United States). The distribution of individual characteristics was determined using simple descriptive statistics. Differences between the distributions of the selected variables were assessed using the chi-square test or Fisher's exact test for categorical data. A univariate analysis of categorical variables was carried out using the chi-square test. Significant variables identified were subsequently included in a binary logistic regression analysis to determine independent predictive variables associated with management changes. All tests were two-tailed, and statistical significance was set at a *P* value of less than .05.

## 3. Results

Archival records from a total of 74 patients (41 women and 33 men) were consecutively included in the current study. The median age was 45.5 years (range 33.7-58.0), and the median duration of disease before MRE was 7.5 years (range 4.7-15.3). Regarding the predominant disease phenotype, 22 patients (29.7%) were classified as inflammatory, 22 (29.7%) as stenosing, and 30 (40.6%) as penetrating. Forty-three patients (58.1%) had ileocolonic disease, 18 (24.3%) had isolated small bowel disease, and 13 (17.6%) had isolated colonic disease. Twenty-seven patients (36.5%) had undergone previous surgery for CD. Of the 74 MREs performed, 19 (25.7%) were considered normal, while 55 (74.3%) had at least one of the nine radiological variables for CD, as previously described. Among the abnormal MREs, 3 patients (5.45%) had solely penetrating disease, 2 patients (13.65%) had a fibrostenosing pattern without signs of active disease, 14 patients (25.45%) had inflammatory disease only, and 36 patients (65.45%) had a mixed pattern.

Table 1 presents the potential associations between demographic, clinical, laboratory, and endoscopic characteristics of the patients, with disease stratification based on MRE patterns. Changes in medical management were recorded in 31 (41.9%) of the 74 patients and were significantly associated with MRE disease patterns (*P* < .001).

The influence of disease stratification based on MRE on medical decisions is shown in Table 2. When considering the penetrating pattern only, significant changes in medical decisions were detected (*P* = .012). All 3 patients with the penetrating phenotype elucidated by MRE had subsequent changes in medical treatment. One patient had treatment optimization, with the initiation of biological therapy, while the other two underwent surgery. The mixed pattern on MRE also had a significant influence on medical decisions (*P* = .024). Among the 36 patients classified as having a mixed pattern, 14 (38.9%) initiated or optimized biological therapy, 3 (8.3%) initiated antibiotics with or without percutaneous drainage, and 4 (11.1%) underwent surgery.

When the MRE results were considered normal, the medical decisions did not change (*P* = .001). Only one of the patients with normal MRE results (5.3%) had a downgrade of the therapeutic regimen. The analysis did not reveal a significant influence of purely inflammatory or fibrostenosing patterns on medical decisions.

The agreement of MRE parameters with clinical, endoscopic, and laboratory values is demonstrated in Table 3. We found significant agreement between the absence of inflammatory criteria on MRE and clinical remission based on the HBI (*P* = .037). Nevertheless, there was no correlation between the parameters of penetrating or fibrostenosing disease on MRE and the HBI. The presence of inflammatory criteria on MRE was significantly correlated with high levels of faecal calprotectin (FC) (*P* = .005). On the other hand, no correlation was found between inflammatory parameters on MRE and other laboratory tests, such as CRP, ESR, and haemoglobin. There was no correlation between inflammatory parameters on MRE and the presence of endoscopic activity on the SES-CD, but the presence of penetrating criteria on MRE was significantly associated with the Simple Endoscopic Activity Score in CD, indicating active disease (*P* = .048). No correlation was found between MRE parameters and the Rutgeerts score. Last, there was significant agreement between the presence of fibrostenosing criteria and a long disease duration (*P* = .027).

In the univariate analysis, only colonoscopy (*P* = .004) and MRE (*P* = .010) were associated with management changes. None of the clinical or laboratory baseline parameters analysed were regarded as significant independent predictors of management changes. In the binary logistic regression analysis, the model consisting of the two variables was able to accurately predict 73% of the management changes. In regard to inflammatory activity, the analysis of MRE combined with colonoscopy had a significant impact on management changes. In particular, when both MRE and colonoscopy were normal (deep healing) or abnormal (no healing), management changes were observed in 14.3% and 67.8% of patients, respectively. On the other hand, when either transmural (MRE) or mucosal (colonoscopy) inflammation was present, management changes occurred in 31.6% and 30.8% of patients, respectively (Table 4).

## 4. Discussion

Until the middle of the last decade, the evaluation of the small bowel in CD was performed by SBFT. Subsequently, CTE and MRE were introduced, and these radiological imaging modalities rapidly became the preferred diagnostic methods for that purpose [24]. In fact, during the first years of use, several studies demonstrated the superiority of CTE and MRE over SBFT for the evaluation of CD [17]. Nonetheless, in addition to having the same accuracy as CTE, MRE has important advantages, such as the absence of ionizing radiation and the best distinction between inflammation and fibrosis [25].

The percentage of patients who had previously undergone surgery in this study is similar to the percentages in some previously reported studies (36.5%). In contrast, the base population analysed here had a high percentage of patients with severe CD phenotypes, as more than two-thirds of patients had penetrating or fibrostenosing disease, which was higher than that in the populations examined in similar studies, which also reflects a relatively large number of patients using biological therapy [26, 27]. As the outpatient clinic in this study is the referral centre for IBD, it is expected that we would have a greater concentration of patients with severe disease. The indications for MRE in this study were broad, with a spectrum ranging from asymptomatic patients in the posttreatment follow-up to patients with active disease based on the HBI and ileocolonoscopy, similar to other retrospective studies [28]. Despite this, the percentage of normal imaging (25.7%) was not higher than that in studies that included only symptomatic patients [20].

The results of this study confirmed that the absence of signs of inflammatory activity on MRE is usually associated with clinical remission based on the HBI. On the other hand, in a more in-depth analysis, we observed that the clinical index, HBI, is not a good predictor of the findings on MRE in asymptomatic patients. In the subgroup of subjects in clinical remission, more than half had at least one radiological criterion of inflammatory activity on MRE. This result appears to reinforce the need for a routine evaluation of luminal and possibly transmural healing by imaging exams during the follow-up of patients. It is already well established in the literature that mucosal healing is associated with a long duration of clinical remission, a long time to clinical relapse, and a decreased need for hospitalization and surgery in CD patients [29, 30]. Takenaka et al. showed good accuracy of the relationship between ulcer healing on MRE, defined by a MaRIA score < 11, and endoscopic healing assessed by balloon-assisted enteroscopy, supporting this radiological method for the indirect investigation of deep healing [31].

The present findings also raise the debate about the importance of monitoring asymptomatic patients with CD to detect subclinical recurrences. Previous studies have indicated that MRE could be useful in asymptomatic patients to predict clinical recurrence. For instance, in a study conducted by Lee et al., active inflammation on MRE increased the risk for clinical relapse (hazard ratio: 6.985; 95% confidence interval, 1.024–47.649) in asymptomatic patients [32]. Naganuma and coworkers showed that ulceration and a MaRIA score ≥ 36.3 were predictive of clinical recurrence. The cut-off value used had a sensitivity of 75% and a specificity of 70% for predicting recurrence [33].

In this study, we demonstrated a significant association between the presence of inflammatory criteria on MRE and high FC values. This is in line with the results of previous prospective studies that showed a good correlation between FC levels and small bowel inflammation on MRE [34–36]. On the other hand, our findings did not show a significant correlation between inflammatory criteria on MRE and CRP levels. Although some previous studies showed a significant correlation between CRP levels and inflammation on MRE, this issue is controversial in the literature [37, 38]. Here, the present findings appear to support the superiority of FC over CRP in the detection of inflammation in patients with CD. Hence, considering that the FC test is easy to perform and has costs less than MRE, we propose that FC could be used as a screening test to define patients with asymptomatic CD who would benefit from MRE in their follow-up. Another possibility would be the use of FC and MRE in an alternate fashion in the follow-up of patients with CD, which would allow the detection of both inflammatory and noninflammatory manifestations, such as stenosis and fistulas, which do not significantly alter FC levels.

In this study, a significant association was detected between the penetrating phenotype in MRE and disease activity based on ileocolonoscopy, as evaluated by the SES-CD. However, no significant association was found between the inflammatory or fibrostenosing criteria in MRE and disease activity based on ileocolonoscopy. These findings differ from those in a retrospective study by Sagami et al., who reported a positive correlation between the SES-CD and the MaRIA scores in patients with inflammatory activity, while no correlation was found in individuals with fibrostenosing or penetrating disease [39]. Although our work presents a relatively small number of patients with exclusively proximal CD, these divergent findings in the literature may be due to a high incidence of terminal ileal disease in topography possibly not accessible by colonoscopy. In addition, we cannot rule out the possibility that MRE may have overestimated the inflammatory process in some cases. In fact, penetrating disease is usually regarded as a more severe CD phenotype, frequently accompanied by associated inflammatory activity, which may explain, at least in part, the association between penetrating disease on MRE and inflammatory activity on ileocolonoscopy. In addition, it is important to call attention to the fact that, in this study, the colon was affected in more than 80% of the patients with the penetrating phenotype. Therefore, it appears that the penetrating phenotype is more likely to be severe and to affect multiple intestinal sites. Moreover, it is interesting to note that 43.2% of patients in this study presented either mucosal healing with transmural inflammation or mucosal inflammation with transmural healing. Such divergence reinforces the importance of combining colonoscopy and MRE in the evaluation and follow-up of patients with CD.

A long disease duration was associated with the presence of fibrostenotic criteria on MRE, a result that is in accordance with data from the literature indicating the natural evolution of the disease with a predominant fibrotic phenotype [40–42]. Nevertheless, the fibrostenotic phenotype was not significantly associated with the HBI or calprotectin level. These results reinforce the importance of the discussion about the need for performing MRE or CTE in the follow-up of asymptomatic or oligosymptomatic patients with long-standing CD. Such patients, especially those with small bowel disease, who present a high risk of progression to the fibrostenosing phenotype, may benefit from serial MRE to detect early abnormalities. This would probably allow the application of more conservative treatments, such as endoscopic balloon dilation or more localized surgical resections.

In contrast to all clinical, laboratory, and endoscopic results, the MRE findings had a significant impact on treatment modification. The most common MRE finding in this study was the mixed pattern (48.64%), in which the patient had radiological criteria of more than one pattern (inflammatory, penetrating, and fibrostenosing). This group is quite heterogeneous, with MRE findings ranging, for example, from mild changes, such as parietal thickening, with slight contrast enhancement in the mucosa to long segments with fixed contrast-enhanced stenosis associated with complex fistulas. A total of 58.3% of patients with the mixed pattern in MRE had a change in clinical treatment. Of these individuals, two-thirds had their pharmacological therapy optimized (almost all with changes to or the onset of biologicals). All of these subgroups had radiological criteria indicating inflammatory activity associated with criteria for penetrating and/or stenosing disease. The remaining one-third of patients underwent antibiotic therapy or surgery. The remaining 41.7% of patients with mixed patterns did not have their treatment altered after MRE. The combination of radiological criteria found in this subgroup was diverse, but none of the patients had an acute complication, such as an abscess. Of the individuals in this subgroup, two-thirds were in clinical remission, having performed the examination for posttreatment evaluation, 20% had mild clinical activity, and 13.33% had moderate clinical activity. One of the possible justifications for not changing the medical treatment in this subgroup of patients, who are mostly asymptomatic or oligosymptomatic, with alterations seen only on MRE, is the limitation of access to the optimization or exchange of biological medications in the health system.

The second most prevalent pattern on MRE in our study was a normal exam. As expected, in light of this result, all patients except for one had their therapy unchanged. The purely penetrating pattern on MRE showed a marked association with changes in medical treatment (surgery or the optimization of drug therapy). In contrast, the pure inflammatory and fibrostenosing patterns were not associated with changes in treatment. Regarding the inflammatory pattern, this finding may be explained, for example, by the great heterogeneity in terms of MRE findings, ranging from few inflammatory signs in localized disease to all four inflammatory criteria in extensive disease. In the case of the exclusively fibrostenosing pattern, the small number of patients in this subgroup renders data on unchanged medical conduct difficult to interpret. Previously, several independent investigations demonstrated the efficacy of MRE as a diagnostic method for the evaluation of the small intestine in CD, but few evaluated its impact on the patient's therapeutic management [20–22, 28, 43] or which radiological abnormalities correlate better with changes in medical decisions [44]. In this respect, studies found in the literature so far are mostly small, retrospective, and unicentric. The present study also has limitations that should be mentioned, including its retrospective design and the basically descriptive nature of the MRE findings, not following known scores and not comparing different MRE scores. However, it is interesting to note that although several clinical, laboratory, and endoscopic methods were considered, changes in therapeutic decisions were mostly based on the MRE findings.

In conclusion, in addition to having a critical role in the diagnosis of CD, which is already well established in the literature, MRE is also important in the follow-up of patients. MRE is capable of detecting inflammatory activity in asymptomatic patients and is a useful resource for the evaluation of deep remission, which is currently the ultimate target of treatment in IBD. The frequency with which MRE should be performed during follow-up was not investigated in this study, and further studies will be needed for that purpose. Finally, MRE constitutes an important noninvasive method in the follow-up of patients with ileocolonic CD due to its significant impact on therapeutic decisions. MRE has the ability to identify complications and to detect abnormalities, even in asymptomatic patients and in those with normal colonoscopies. Further prospective studies will be necessary to determine the best intervals for applying MRE on a routine basis.

## Figures and Tables

**Table 1:** Characteristics of the patients and disease stratification based on magnetic resonance enterography.

Baseline characteristic	All patients (*n* = 74)	Inflammatory pattern (*n* = 14)	Penetrating pattern (*n* = 3)	Fibrostenosing pattern (*n* = 2)	Mixed pattern (*n* = 36)	No abnormality (*n* = 19)	*P* value
Age, median (years)	45.5 (33.7-58)	45.5 (38.2-59.3)	56 (16-)	51 (45-)	51 (30.2-58)	41 (23-55)	0.154
Disease duration (years)	7.5 (4.7-15.3)	5.0 (3.7-15)	6.0 (0-)	14.5 (12-)	9.5 (5.2-21.5)	6.0 (3-10)	0.091
Females	41 (55.4%)	7 (50.0%)	1 (33.3%)	2 (100.0%)	21 (58.3%)	10 (52.6%)	0.781
Age at diagnosis (years)							
A1 (<17)	6 (8.1%)	0 (0.0%)	1 (33.3%)	0 (0.0%)	1 (2.8%)	4 (21.1%)	0.350
A2 (17–40)	42 (56.8%)	9 (64.3%)	0 (0.0%)	2 (100.0%)	23 (63.9%)	8 (42.1%)	
A3 (>40)	26 (35.1%)	5 (35.7%)	2 (66.7%)	0 (0.0%)	12 (33.3%)	7 (36.8%)	
Behaviour							
B1 (inflammatory)	22 (29.7%)	7 (50.0%)	0 (0.0%)	0 (0.0%)	6 (16.7%)	9 (47.4%)	0.109
B2 (stricturing)	22 (29.7%)	4 (28.6%)	0 (0.0%)	2 (100.0%)	12 (33.3%)	4 (21.1%)	
B3 (penetrating)	30 (40.5%)	3 (21.4%)	3 (100.0%)	0 (0.0%)	18 (50.0%)	6 (31.6%)	
Location							
L1 (ileal)	18 (24.3%)	4 (28.6%)	0 (0.0%)	1 (50.0%)	9 (25.0%)	4 (21.1%)	0.452
L2 (colonic)	13 (17.6%)	1 (7.1%)	1 (33.3%)	0 (0.0%)	4 (11.1%)	7 (36.8%)	
L3 (ileocolonic)	43 (58.1%)	9 (64.3%)	2 (66.7%)	1 (50.0%)	23 (63.9%)	8 (42.1%)	
Perianal disease	16 (21.6%)	3 (21.4%)	3 (100.0%)	0 (0.0%)	7 (19.4%)	3 (15.8%)	0.481
Proximal disease	6 (8.1%)	1 (7.1%)	0 (0.0%)	0 (0.0%)	4 (11.1%)	1 (5.3%)	0.604
Previous surgery	27 (36.5%)	4 (28.6%)	0 (0.0%)	0 (0.0%)	13 (36.1%)	10 (52.6%)	0.092
EIM	14 (18.9%)	1 (7.1%)	2 (66.7%)	1 (50.0%)	9 (25.0%)	1 (5.3%)	0.080
Smoking history	13 (17.6%)	2 (14.2%)	0 (0.0%)	0 (0.0%)	6 (16.7%)	5 (26.3%)	0.251
HBI							
Activity	28 (37.8%)	6 (42.9%)	1 (33.3%)	1 (50.0%)	16 (44.4%)	4 (21.1%)	0.082
Remission	46 (62.2%)	8 (57.1%)	2 (66.7%)	1 (50.0%)	20 (55.6%)	15 (78.9%)	
CRP (mg/L)	2.8 (1.4-5.5)	2.6 (1.3-3.0)	1.1 (1.0-)	4.3 (4.3-)	3.6 (2.1-6.0)	2.3 (0.5-16.3)	0.772
ESR (mm/h)	28 (16-41)	37 (20-56)	25 (17-)	41 (41-)	28 (15-40)	19 (10-35)	0.467
Haemoglobin (g/dL)	13.0 (11.5-13.7)	12.1 (9.8-13.3)	12.5 (11-)	13.0 (13.0-)	12.9 (11.5-13.8)	13.3 (12.8-13.8)	0.205
Calprotectin (*μ*g/g)	177 (83-492)	201 (110-299)	55 (30-)	72 (72-)	299 (98-997)	100 (20-180)	0.238
Colonoscopy scores							
SES-CD (*n* = 55)	4 (1-10)	3.5 (1-11.3)	9 (2-)	5 (4-)	6.5 (1.0-12.3)	3.5 (0-7.8)	0.297
Rutgeerts score (*n* = 19)	1 (0-3)	2 (0.25-3.0)	—	—	2 (0-3.0)	0 (0-2.5)	0.390
Treatment							
IMM only	47 (63.5%)	10 (71.4%)	3 (100.0%)	2 (100.0%)	21 (58.3%)	11 (57.9%)	0.561
Biologics (±IMM)	27 (36.5%)	4 (28.6%)	0 (0.0%)	0 (0.0%)	15 (41.7%)	8 (42.1%)	
Change in management	31 (41.9%)	6 (42.9%)	3 (100.0%)	0 (0.0%)	21 (58.3%)	1 (5.3%)	<0.001

Values are presented as the median with the interquartile range or as *n* with the percentage; EIM: extraintestinal manifestation; HBI: Harvey and Bradshaw index; CRP: C-reactive protein; ESR: erythrocyte sedimentation rate; SES-CD: scoring endoscopic system for Crohn's disease; IMM: immunomodulator.

**Table 2:** Influence of disease stratification based on magnetic resonance enterography in medical decisions.

*Exclusive MRE pattern*	*Medical decision*					*P* value
	No change	Biological therapy optimization/initiation	Downstaging therapy	Antibiotics/percutaneous drainage	Surgery	
Inflammatory (*n* = 14)	8 (57.1%)	5 (35.7%)	0 (0.0%)	0 (0.0%)	1 (7.1%)	0.824
Penetrating (*n* = 3)	0 (0.0%)	1 (33.3%)	0 (0.0%)	0 (0.0%)	2 (66.7%)	0.012
Fibrostenosing (*n* = 2)	2 (100.0%)	0 (0.0%)	0 (0.0%)	0 (0.0%)	0 (0.0%)	0.830
Mixed (*n* = 36)	15 (41.7%)	14 (38.9%)	0 (0.0%)	3 (8.3%)	4 (11.1%)	0.024
Normal (*n* = 19)	18 (94.7%)	0 (0.0%)	1 (5.3%)	0 (0.0%)	0 (0.0%)	0.001
Total (*n* = 74)	43 (58.1%)	20 (27.0%)	1 (1.3%)	3 (4.0%)	7 (9.5%)	

MRE: magnetic resonance enterography. The MRE patterns analysed are mutually exclusive. Values are presented as numbers, with percentages in parentheses.

**Table 3:** Agreement of magnetic resonance enterography parameters with selected clinical, endoscopic, and laboratory variables.

Patient variable	*MRE patterns*								
	*Inflammatory*			*Penetrating*			*Fibrostenosing*		
	Absent (*n* = 27)	Present (*n* = 47)	*P* value	Absent (*n* = 52)	Present (*n* = 22)	*P* value	Absent (*n* = 43)	Present (*n* = 31)	*P* value
HBI ≤ 4 (*n* = 46)	21 (77.8%)	25 (53.2%)	0.037	34 (65.4%)	12 (54.5%)	0.383	28 (65.1%)	18 (58.1%)	0.540
HBI > 4 (*n* = 28)	6 (22.2%)	22 (46.8%)		18 (34.6%)	10 (45.5%)		15 (34.9%)	13 (41.9%)	
Disease duration (years)	7 (3-12)	9 (5-20)	0.278	7 (4-12)	10 (5.8-25.8)	0.107	6 (4-11)	12 (6-20)	0.027
CRP (mg/L)	2.1 (1.0-6.1)	3 (1.9-5.7)	0.202	2.9 (1.4-5.2)	2.6 (1.3-13.4)	0.789	2.3 (1.2-7.8)	3.6 (2.1-6.1)	0.335
ESR (mm/h)	19 (10-35)	30 (19-45)	0.128	29 (16-42)	24 (12-35)	0.466	21 (16-38)	30 (17-42)	0.514
Haemoglobin (g/dL)	13.4 (12.7-13.7)	12.3 (11.4-13.9)	0.215	12.9 (11.5-13.6)	13.6 (11.4-14.1)	0.150	13.1 (11.6-13.7)	13 (11.5-14)	0.924
Calprotectin (*μ*g/g)	76 (28-171)	255 (107-618)	0.005	183 (107-490)	89 (79-932)	0.885	142 (43-201)	288 (88-965)	0.105
SES-CD	4 (1-9.3)	4 (1.5-11.5)	0.474	3 (0.5-8.5)	8 (2.8-13)	0.048	4.5 (1.8-10)	4 (1-10.5)	0.876
Rutgeerts	0 (0-2.5)	2 (0-3)	0.336	1 (0-3)	2.5 (1.3-3)	0.177	1 (0-3)	2 (0-3)	0.671

MRE: magnetic resonance enterography. The MRE patterns analysed are not mutually exclusive. Values are presented as numbers, with percentages in parentheses, or as the median with the interquartile range; HBI: Harvey and Bradshaw index; CRP: C-reactive protein; ESR: erythrocyte sedimentation rate; SES-CD: scoring endoscopic system for Crohn's disease; Rutgeerts: scoring endoscopic system for postoperative patients with Crohn's disease.

**Table 4:** Impact of inflammatory activity based on magnetic resonance enterography and colonoscopy on management changes.

Management	All patients (*n* = 74)	Deep healing (*n* = 14)	Mucosal healing/transmural inflammation (*n* = 19)	Transmural healing/mucosal inflammation (*n* = 13)	No healing (*n* = 28)	*P* value
Change	31 (41.9%)	2 (14.3%)	6 (31.6%)	4 (30.8%)	19 (67.8%)	0.003
No change	43 (58.1%)	12 (85.7%)	13 (68.4%)	9 (69.2%)	9 (32.2%)	

Deep healing was defined by the absence of mucosal or transmural inflammation; no healing was defined by the presence of both mucosal and transmural inflammation.

## Data Availability

The raw data supporting the conclusions of this manuscript will be made available by the authors, without undue reservation, to any qualified researcher.
